# Carcinoma arising within sciatic nerve endometriosis: a case report

**DOI:** 10.1093/jscr/rjab512

**Published:** 2021-12-11

**Authors:** Adaiah Yahaya, Govind Chauhan, Adeyemi Idowu, Vaiyapuri Sumathi, Rajesh Botchu, Scott Evans

**Affiliations:** GPST2, Leach Heath Medical Practice, Birmingham, UK; Specialty Registrar (ST7), Trauma and Orthopaedics, Royal Orthopaedic Hospital, The Woodlands, Birmingham, UK; Speciality Trainee, Histopathology, Royal Orthopaedic Hospital, Birmingham, UK; Royal Orthopaedic Hospital, The Woodlands, Birmingham, UK; Royal Orthopaedic Hospital, The Woodlands, Birmingham, UK; Royal Orthopaedic Hospital, The Woodlands, Birmingham, UK

## Abstract

Endometriosis is a common condition with significant morbidity. There have been case reports of endometrial deposits affecting the sciatic nerve. Sciatic nerve endometriosis presents with cyclical sciatica and is often difficult to diagnose as it mimics many other causes of sciatica. We report the first case of histological proven endometrial carcinoma arising in a pre-existing sciatic nerve endometriosis. This was initially managed with radiotherapy however symptoms persisted and she opted to have surgery with the aim of better symptom control and long-term prognosis.

## INTRODUCTION

Endometriosis affects 10% of women of reproductive age [1] and is a cause of chronic pain and significant morbidity. Endometriosis can be intrapelvic or extrapelvic. Common sites of endometriosis are the pelvic peritoneum, rectovaginal septum and ovaries. Retrograde menstruation coupled with immune dysfunction and genetic mutations are plausible aetiological factors of endometriosis. Wherever the endometriotic deposits are, a common pathogenesis in the accompanying chronic cyclical pain and debility is angiogenesis, adhesions and fibrosis with scarring, neuronal infiltration and anatomical distortion [2].

## CASE REPORT

### Presentation

A female in her late 1950s was referred to the orthopaedic oncology clinic with an 18 month history of left lower limb sciatica. She also complained of progressive weakness in her left leg, decreased sensation on the lateral aspect of the leg and weakness and sensory loss in her foot. She had urinary urgency, but no frequency or incontinence, with some paraesthesia in her labia, but no objective sensory loss in the perianal or genital area.

The severity of her pain significantly impaired her quality of life, and she required regular analgesia with Tramadol, Gabapentin, Dihydrocodeine and Naproxen to ease her symptoms. She has a background medical history of asthma and newly diagnosed type 2 diabetes mellitus. She smokes 20 cigarettes per day.

On examination, she had a body mass index of 31. There was no active dorsiflexion or plantar flexion of any toes or ankle. Her knee flexion was MRC grade 4/5, compared to extension of 5/5. She had sensory loss in the foot and the lateral lower leg, but had intact sensation over the medial aspect of the lower leg and above her knee. Perianal sensation was normal. There were no associated cafe au lait spots.

**Figure 1:**
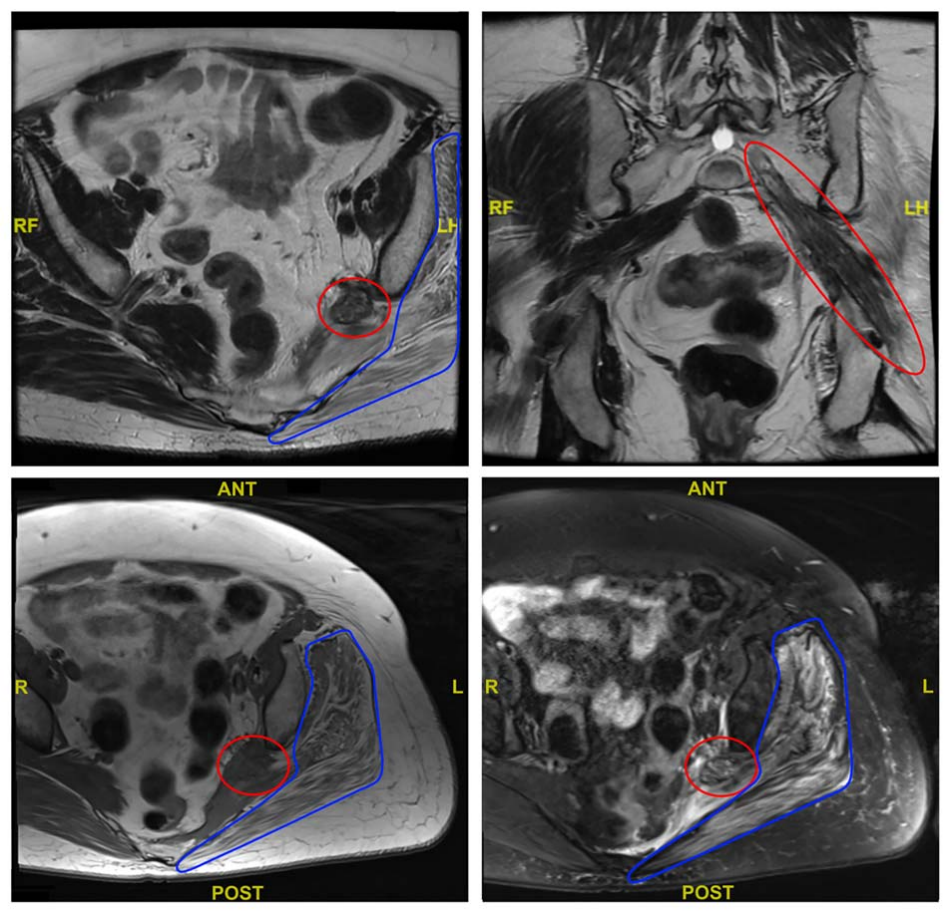
Selected axial and coronal views from MRI pelvis showing a thickened and diseased left sciatic nerve (red circle) and atrophied left glutei with fatty infiltration (blue outline).

An magnetic resonance imaging (MRI) pelvis was performed prior to referral, which showed a thickened and inflamed left sciatic nerve as it emerged from the sacral foramen and continued through the sciatic notch. This was associated with marked oedema and fatty atrophy of the left piriformis, glutei (gluteus maximus, gluteus medius and gluteus minimus) and obturator internus. Selected views from the MRI are shown in Fig. 1.

The initial suspicion was likely to be either a benign nerve sheath tumour growing within the sciatic nerve causing a loss of function, or potentially a malignant tumour known as a malignant peripheral nerve sheath tumour.

### Investigations

Further investigations were needed to confirm the diagnosis, and after a multidisciplinary team discussion (MDT), she was referred for a whole body PET computed tomography (CT) scan and a CT-guided biopsy.

**Figure 2:**
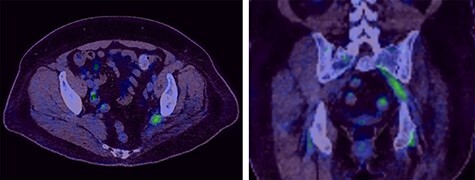
Selected axial and coronal slices from PET scan showing increased activity in the left sciatic nerve.

The PET CT scan findings were thickening of the left intra-pelvic portion of the sacral plexus (L5 and S1 nerve roots) up to the level of greater sciatic notch with associated increased activity as seen in Fig. 2.

She underwent a CT-guided biopsy of the thickened sciatic nerve.

**Figure 3:**
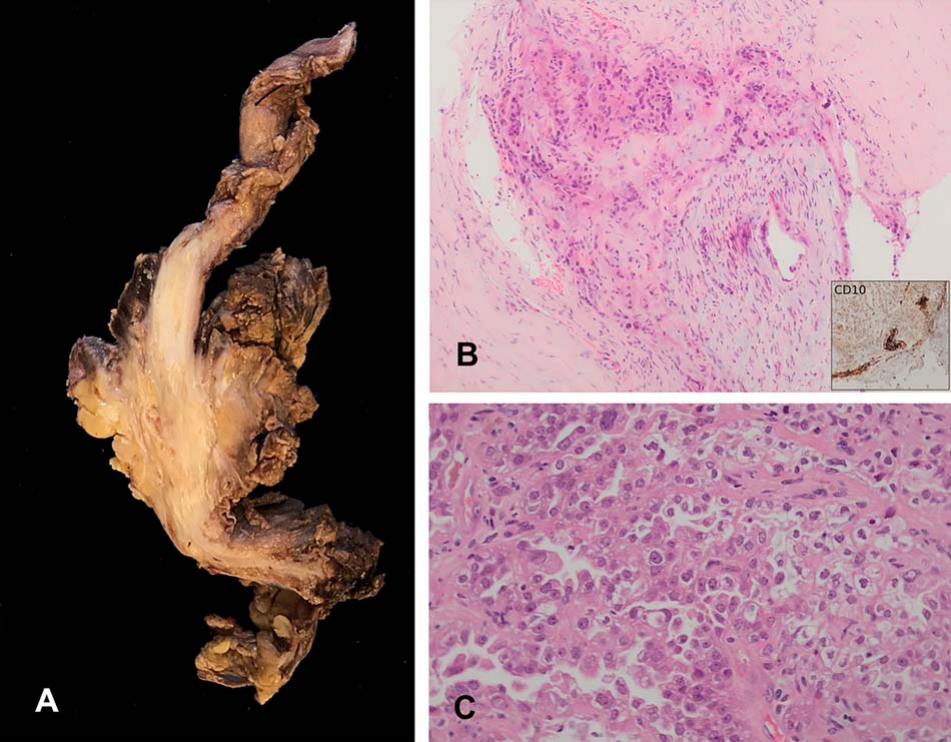
Histological specimen. (**A**) Main tumour dissected out shows extensive infiltration of the sciatic nerve. (**B**) Endometriotic focus within the tumour, endometrial stroma highlighted by immunohistochemical marker CD10. (**C**) The tumour showed features of endometrial carcinoma with glandular, papillary and solid pattern.

The sciatic nerve biopsy showed nerve fibres infiltrated by epithelial cells featuring enlarged hyperchromatic nuclei, prominent nucleoli and clear to pale eosinophilic cytoplasm. These cells were highlighted with oestrogen receptor and PAX8 immunohistochemical markers. Focal areas of glandular differentiation were noted. There were some hyperchromatic spindle cells focally highlighted with CD10, suggestive of endometrial stroma (Fig. 3).

In the absence of a clinical and CT-TAP evidence of any neoplastic lesion, the lesion was considered as a secondary malignancy arising in an endometriotic foci.

Subsequently, histological examination of the tumour from the amputation specimen confirmed the diagnosis of clear cell carcinoma arising in a foci of endometriosis. This was confirmed with positive immunomarker with Napsin A.

Epithelial differentiation in soft tissue sarcomas such as biphasic synovial sarcoma, myoepithelioma, malignant peripheral nerve sheath tumour and epithelioid sarcoma were excluded. Fluorescence-*in-situ* hybridization test did not reveal SS18 gene rearrangement [3, 4].

### Differential diagnosis

In addition to a benign or malignant nerve sheath tumour, other conditions with similar presentation include lymphoma, amyloidosis and metastatic carcinomas to the sciatic nerve.

### Treatment

The case was discussed at the gynaecology and sarcoma MDTs, and a diagnosis of endometrial carcinoma infiltrating the sciatic nerve in a focus of previous endometriosis was agreed upon.

The gynaecology MDT recommended radiotherapy, which was delivered as a radical treatment dose of 55GY in 25 fractions. Unfortunately, the patient’s symptoms did not improve, with worsening pain and deterioration of function in her left lower limb. A non-surgical approach to pain management was discussed, including nerve blocks and nerve stimulators, but the patient requested surgical management. Furthermore, the tumour was non-metastatic and was not controlled by radiotherapy hence surgery was deemed appropriate. The options of a left hemi-sacrectomy and excision of sciatic nerve, or a hindquarter amputation given previous radiotherapy were discussed. A hindquarter amputation was deemed to offer the patient the best oncological management given that the patient’s disease was localized to the nerve (non-metastatic). Limb salvage surgery was discussed with the patient. However, the patient did not wish to proceed with this because of the possibility of compromised resection margins and the inherent risks of wound complications and/or pelvic visceral injury operating within a post-radiotherapy field.

## DISCUSSION

Endometriosis affects 5–10% of women in the reproductive age group [5]. A systematic review of extra pelvic endometriosis over the past 20 years found 12 case reports and case series of extra pelvic nerve or muscle involvement [6]. A 2021 survey of British gynaecological endoscopists found the annual incidence of thoracic endometriosis to be about 245–545 cases [7]. A prospective case series report from 2004 to 2016, reported 259 cases of endometriosis with intra- and extra-abdominal endometriosis, including involvement of the sciatic nerve [8]. The 67% of reported cases of sciatic nerve endometriosis occur on the right, in contrast to intrapelvic endometriosis, which occurs more on the left and provides evidence in support of the retrograde menstruation theory [9].

Isolated sciatic nerve endometriosis has also been reported. One such report is in a 24-year-old with right sciatic nerve endometriosis treated laparoscopically [10]. An example of a left sciatic nerve endometriosis is that reported in a 25-year-old with a history of constant pain in the thigh of 2 months prior to presenting to her GP. She was ultimately referred to the orthopaedic team after not responding to anti-inflammatory medication and physiotherapy, where a histological diagnosis was made, and she had microsurgical dissection of the endometrial tissue from the sciatic nerve with good post-operative outcome [11].

The mean interval of diagnosis of sciatic nerve endometriosis from onset of symptoms is reported as 3.7 years [12]. There is a single case report of low grade endometrial stromal sarcoma arising from sciatic nerve endometriosis in the literature. However, the patient had extensive intra-pelvic endometriosis in addition to involvement of the sciatic nerve [13].

Symptoms of sciatic nerve endometriosis might persist even with control of pelvic endometriosis. The use of medical treatment has been demonstrated to be unsuccessful in controlling symptoms of sciatica in sciatic nerve endometriosis [2, 10]. The previously reported sciatic nerve endometrial stromal sarcoma was extensive, with tumour extension to bowel and bladder and hence surgically unrespectable. There was initial mid-treatment response to multi-agent chemotherapy but the patient deteriorated and palliative radiotherapy offered partial symptom control [13]. The patient in our report had a resectable tumour with symptoms that did not improve with radiotherapy, and hence surgery offered the best chance of cure and symptom control. Furthermore, in the presence of features of nerve damage, surgery is the preferred option [10].

Endometriosis of the sciatic nerve remains a rare condition, and malignant transformation within a sciatic nerve deposit has only been reported once previously. This highlights the difficulty in managing this lady and the need for a multidisciplinary approach as adopted in this case. It also emphasizes the need for an individualized approach aimed at disease control/clearance and relief of symptoms.

## CONFLICT OF INTEREST STATEMENT

The authors have no conflict of interests to declare.
